# Response to daratumumab-retreatment in patients with multiple myeloma

**DOI:** 10.1007/s00277-024-05991-7

**Published:** 2024-09-23

**Authors:** Laura Souren, Gabriele Ihorst, Christine Greil, Monika Engelhardt, Ralph Wäsch

**Affiliations:** 1 Department of Hematology, Oncology and Stem Cell Transplantation, Faculty of Medicine, Medical Center, University of Freiburg, Hugstetterstrasse 55, 79106 Freiburg, Germany; 2 Comprehensive Cancer Center Freiburg (CCCF), Faculty of Medicine, Medical Center, University of Freiburg, Freiburg, Germany; 3 Clinical Trials Unit, Faculty of Medicine, Medical Center, University of Freiburg, Freiburg, Germany

**Keywords:** CD38-antibody, Retreatment, Multiple myeloma

## Abstract

Daratumumab is an effective therapy in multiple myeloma (MM). We assessed whether daratumumab retreatment may re-induce significant responses and which patients do benefit the most. We hypothesized, that there is effective synergism between daratumumab and alternating antimyeloma drug combinations during retreatment and that retreatment is safe and effective. Here, we analyzed 293 consecutive MM patients receiving daratumumab at our institution from 2016 until 2023 retrospectively, and compared responses, side effects and survival of the first daratumumab treatment line and its retreatment. We identified 22/293 (8%) patients with daratumumab retreatment. These patients showed an advanced age and ISS/R-ISS stages, and ≥ 3 lines of prior antimyeloma therapy in 91%. Of note, the median durations of the first and subsequent daratumumab treatment were similarly long. We confirmed a therapy break between daratumumab lines as advantageous. Daratumumab retreatment was effective, with responses declining only gradually from its first use to subsequent first and second retreatment with 64%, 46% and 43%, respectively. Interestingly, comparable progression free survival rates were observed with 11.5, 12 months and not reached, respectively. Consistently, adverse events per daratumumab line did not increase. Our findings suggest that well-selected daratumumab-exposed MM patients may show rewarding responses to daratumumab retreatment, the more with alternating antimyeloma combinations, initial good response and CD38-antibody-treatment pauses, thereby proving CD38-antibody-retreatment as feasible, effective and non-toxic. Confirmatory studies are required to further validate our results.

## Introduction

Multiple myeloma (MM) is a malignant hematologic neoplasia characterized by bone marrow infiltration of monoclonal plasma cells. The secreted functionless monoclonal immunoglobulins or light chains can be detected as the M-protein and clonally increased light chains in serum and/or urine [1, 2]. MM remains an incurable disease, although the introduction of new classes of drugs contributed to deeper remissions and the significant improvement in survival over the past decades [3–5]. Nevertheless, relapses may typically occur during the course of the disease and require new therapeutic approaches to regain disease control [6–8]. In 2015, monoclonal antibodies were first approved for the treatment of MM and have become well-established therapeutic agents for the disease [9]. 

Daratumumab is a human IgG1κ monoclonal antibody that targets the CD38-antigen, a transmembrane glycoprotein increasingly expressed on myeloma cells [10, 11]. Daratumumab binds with high affinity to a specific epitope on CD38 and leads to apoptosis of CD38 expressing myeloma cells by several mechanisms of action. These comprise direct apoptosis by means of cross-linking, immune-mediated effects including complement-dependent cytotoxicity (CDC), antibody-dependent cell-mediated cytotoxicity (ADCC) and antibody-dependent cellular phagocytosis (ADCP). Immunomodulatory pathways attenuate the immunosuppressive activity of CD38-cells and promote T-cell expansion [11–14]. Various daratumumab-based combinations have been approved for transplant-eligible (TE) and -ineligible (TIE) patients both in newly diagnosed (NDMM) and relapsed/refractory MM (RRMM) [15–17]. 

To further maintain disease control, prior studies had demonstrated that myeloma patients can benefit from retreatment strategies with drugs of the same class (like immunomodulatory agents (IMiDs) or proteasome inhibitors (PIs)) they received in previous treatment lines [18, 19]. Since the CD38-antibody daratumumab is frequently used in everyday clinical practice and in earlier lines, understanding the role of retreating patients with daratumumab has high priority, albeit another CD38-antibody is already available with isatuximab and claims to be feasible and effective after daratumumab treatment. However, a prospective phase 2 study in 32 patients refractory to daratumumab with use of isatuximab monotherapy has shown no objective response rate (ORR), with one minimal response (MR) and stable disease (SD) in 17 patients, with a disease control rate (DCR, defined as ≥ MR or SD ≥ 8 weeks) of 38% [20]. Another retrospective single center analysis in 43 daratumumab-refractory patients, employing daratumumab retreatment in different combinations demonstrated an ORR of 49% [21]. Therefore, to gain further insight into the important question, whether retreatment with daratumumab is feasible, effective and how this should be best performed, we conducted this detailed analysis. We assessed the number of patients at our institution receiving daratumumab as an initial treatment line and then again for retreatment, evaluated their response, side effects and survival. We hypothesized that there is effective synergism between daratumumab and different combination partners during retreatment and that retreatment is possible, effective and safe.

## Methods

### Trial design and oversight

In this single center study, we retrospectively analyzed data obtained from patients with MM who were treated with daratumumab at our institution from May 2016 through January 2023. From initially 293 patients, who fulfilled the inclusion criteria, 39 (13%) were identified, who received multiple (*≥* 2) daratumumab treatment lines. Of these, 17 had to be excluded, not fulfilling daratumumab retreatment criteria defined as administration with another combination partner or after discontinuation of therapy. Twenty-two patients (8%) were considered as having received a daratumumab retreatment and were fully assessed within this study.

The study is registered at Freiburg Register of Clinical Trials under protocol FRKS004511 and was approved by the Ethics Committee of the University of Freiburg (EV 23-1110_1-S1). It was performed according to the guidelines of the Declaration of Helsinki and Good Clinical Practice. All patients gave their written informed consent for institutionally initiated research studies in accordance with the institutional review board guidelines.

### Data analysis/ patients

All disease-specific data were collected using the clinic’s medical information and documentation system. For exact data acquisition, external and internal physicians’ reports and file notes, tumor board protocols, therapy protocols and laboratory results were all thoroughly and repeatedly evaluated. A variety of patient- and disease-related data was collected. Patient demographics were reported including age and date at initial diagnosis (ID), gender and date of death or last seen alive. MM-specific data capturing myeloma type were used. Patient clinical characteristics recorded included risk-scores to capture disease prognosis, as International Staging System (ISS), Revised (R-) ISS, Revised Myeloma Comorbidity Index (R-MCI) [22, 23]. Treatment data were described as the total number of therapy lines patients received, the time between daratumumab lines, whether a different therapy was given in between, the number of daratumumab cycles of the respective lines and whether daratumumab was administered in combination with other standard therapies or as monotherapy. The type of combined agent was recorded and assigned to a group (PI, IMiD, alkylating agent, other). Additionally, it was documented whether patients received a stem cell transplantation (SCT). Adverse events were graded according to the National Cancer Institute`s Common Terminology Criteria for Adverse Events (CTCAE V.5.0).

Thereupon, patients were divided into first daratumumab treatment (D1), first daratumumab retreatment (D2) and second daratumumab retreatment (D3) subgroups. Daratumumab lines in terms of treatment duration and response were assessed and compared according to the International Myeloma Working Group (IMWG) remission criteria.

### Statistical analysis

Primary endpoint was progression-free survival (PFS), which was defined as the time from start of daratumumab treatment until disease progression or until end of daratumumab treatment (censored observation) within this daratumumab line. Deaths under daratumumab treatment did not occur. However, two patients were switched to best supportive care after progression and died thereafter. Secondary endpoints included response rate (ORR was defined as MR or better according to IMWG response criteria) and duration of response (DOR), as well as adverse events (AE).

Data were analyzed using SAS 9.4 statistical software (SAS Institute, Inc, Cary, North Carolina). PFS probabilities were estimated using the Kaplan-Meier method. Comparisons of patients concerning first-line or later-line PFS were conducted visually and descriptively.

## Results

### Patient characteristics

Of 293 patients treated with daratumumab at our institution (D1), 22 patients received daratumumab as retreatment (D2), with 7 patients obtaining a second daratumumab retreatment (D3).

Baseline characteristics of D2 and D3 patients are displayed in Table 1. The median age at ID was 59 years (range, 40–82). At the end of data collection, 91% of patients were still alive, while 9% had died. Men predominated with 64%. The cohort showed frequencies of IgG-MM in 41%, of IgA-MM in 27% and light-chain (LC)-only MM in 32%. Staging according to the ISS was relatively balanced with stage I in 27%, stage II in 32% and stage III in 41%. According to the R-ISS, stage II predominated with almost half (46%). Most patients belong to the R-MCI intermediate-fit stage with 59% in line with prior results [22, 23]. At least one autologous stem cell transplantation (auto-SCT) had been performed in 86% and 36% had also received an allogeneic SCT (allo-SCT). Most of the cohort (91%) had received intensive anti-myeloma treatment with ≥ 3 lines (range 3–7).

**Table 1:** Baseline characteristics of the total cohort (D2 + D3)

	All patients *n* = 22
Demographic data	
Median age at ID [years] (range)	59 (40–82)
Gender: male / female n (%)	14 / 8 (64 / 36)
Patients alive yes / no (%)	20 / 2 (91 / 9)
**Type of MM n (%)**	
IgG / IgA	9 (41) / 6 (27)
LC only	7 (32)
Kappa / lambda	11 (50) / 11 (50)
**Risk-score n (%)**	
ISS stage	
I / II / III	6 (27) / 7 (32) / 9 (41)
R-ISS stage	
I / II / III	4 (18) / 10 (46) / 8 (36)
R-MCI	
Fit (0–3) / intermediate-fit (4–6) / frail (7–9)	7 (32) / 13 (59) / 2 (9)
**Therapy / Outcome n (%)**	
Therapy lines 2 / ≥3 [range]	2 / 20 [3–7] (9 / 91)
SCT yes / no	19 / 3 (86 / 14)
Auto- / + allo-SCT	19 / 8 (86 / 36)

*Abbreviations* ID: initial diagnosis, MM: multiple myeloma, LC: light chains, ISS: International Staging System, R-ISS: Revised International Staging System, R-MCI: Revised Myeloma Comorbidity Index, SCT: stem cell transplantation, auto: autologous, allo: allogeneic

### Treatment

Table 2 summarizes treatment- and outcome-data of the individual daratumumab lines. Patients with D1 received a median of 1 previous line of therapy (LOT, range 1–4), in D2 a median of 3 (range 2–6) prior LOT and in D3 a median of 4 (range 3–7) prior LOT had been performed.

**Table 2:** Therapy and outcome data for respective daratumumab lines

	First daratumumab treatment (D1, *n* = 22)	1. daratumumab retreatment (D2, *n* = 22)	2. daratumumab retreatment (D3, *n* = 7)
**Median line of therapy (range)**	2 (1–4)	4 (2–6)	5 (3–7)
**Median number of daratumumab-cycles (range)**	9 (1–33)	11 (1–46)	8 (1–14)
**Median duration (range) [weeks]**	35 (4-126)	42 (2-146)	30 (3–53)
**Combination-/ Monotherapy** **(%)**	19 / 3 (86 / 14)	20 / 2 (91 / 9)	7 / 0 (100 / 0)
**Dara-based therapy (%)**			
Dara	3 (14)	2 (9)	0 (0)
Dara/PI	16 (72)	9 (41)	4 (57)
Dara/IMID	3 (14)	5 (23)	1 (14)
Dara/other	0 (0)	6 (27)	2 (29)
**Median time interval between (range) [weeks]**			
D1 and D2 (*n* = 22)	34 (1-131)		
D2 and D3 (*n* = 7)	32 (1-109)		
**Therapy line in between yes/no (%)**			
D1 and D2 (*n* = 22)	9 / 13 (41 / 59)		
D2 and D3 (*n* = 7)	2 / 5 (29 / 71)		
**Response n (%)**			
**ORR**	14 (64)	10 (46)	3 (43)
sCR	0 (0)	1 (5)	1 (14)
CR	0 (0)	0 (0)	0 (0)
vgPR	6 (27)	3 (14)	0 (0)
PR	7 (32)	6 (27)	2 (29)
MR	1 (5)	0 (0)	0 (0)
SD	8 (36)	10 (45)	4 (57)
PD	0 (0)	2 (9)	0 (0)
**PFS**			
Median PFS [months]	11.5 (95% CI: 5.1–27)	12 (95% CI: 3.4- NR)	NR (95% CI: 2.8- NR)
6-months-PFS [%]	77 (95% CI: 56.3–96.8)	72 (95% CI: 51.3–93.5)	83 (95% CI: 53.5–100)
12-months-PFS [%]	42 (95% CI: 13.2–70.3)	45 (95% CI: 20.1–70.2)	NR
24-months-PFS [%]	28 (95% CI: 0- 57.1)	45 (95% CI: 20.1–70.2)	--

*Abbreviations* Dara: daratumumab, PI: proteasome inhibitor, IMID: immunomodulatory drug, ORR: overall response rate, sCR: stringent complete remission, CR: complete remission, vgPR: very good partial remission, PR: partial remission, MR: minor response, SD: stable disease, PD: progressive disease, PFS: progression- free survival, CI: confidence interval, NR: not reached

Of note, a similar median number of daratumumab cycles were performed in D1, D2 and D3 with a median of 9, 11 and 8 cycles, respectively. Also, the treatment duration was similar in all daratumumab treatments, with 35, 42 and 30 weeks, respectively.

Most patients of the cohort received daratumumab in combination with another therapeutic agent in all daratumumab treatment-lines (86% vs. 91% vs. 100%, respectively). This was most often performed with a PI in D1, D2 and D3 groups with 72%, 41% and 57%, respectively. Here, bortezomib was a lead combination drug in all daratumumab lines with 64%, 27% and 14%, respectively. More precisely, bortzomib-dexamethasone (Vd) was added to daratumumab in 45%, 18% and 14%, respectively. Also IMiDs were frequently combined with daratumumab, with highest frequencies in D2 with 23% and in the D1 and D3 with 14% each, with lenalidomide (R) dominating. In D1 and in D2, daratumumab was occasionally administered as a monotherapy. In D2 (27%) and D3 (29%), different (other) combination partners were also used. Median interval between D1 and D2 as well as D2 and D3 were similar with 34 and 32 weeks, respectively.

**Fig. 1:**
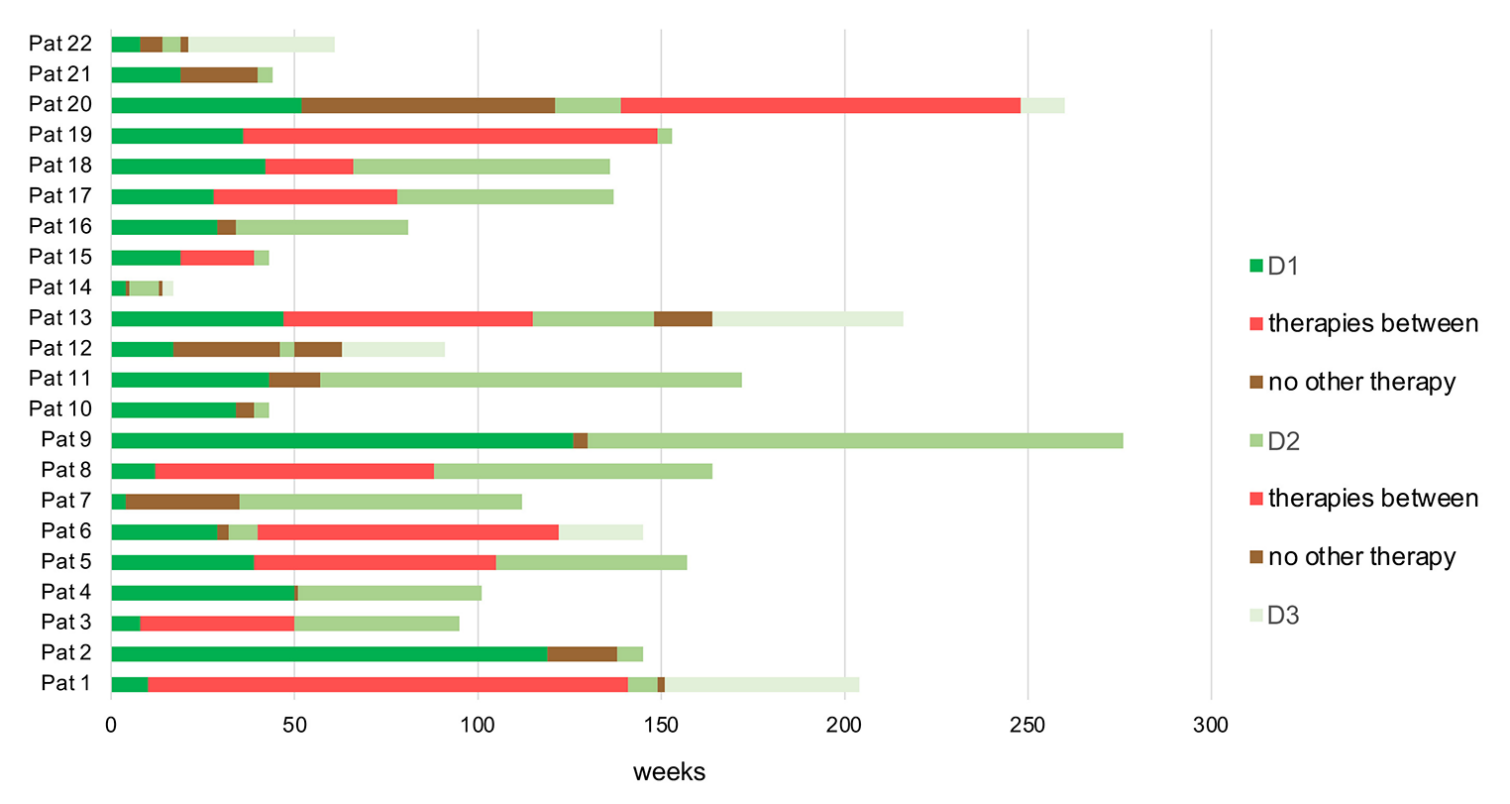
Lengths of daratumumab lines and treatment breaks. D1 = first daratumumab treatment, D2 = first daratumumab retreatment, D3 = second daratumumab retreatment

Those who did not receive intermediate daratumumab-free LOTs, but a treatment break without therapy between two daratumumab lines dominated in all lines with 59% and 71%, respectively. The duration of daratumumab lines and treatment breaks are illustrated in Fig. 1.

### Efficacy

**Fig. 2:**
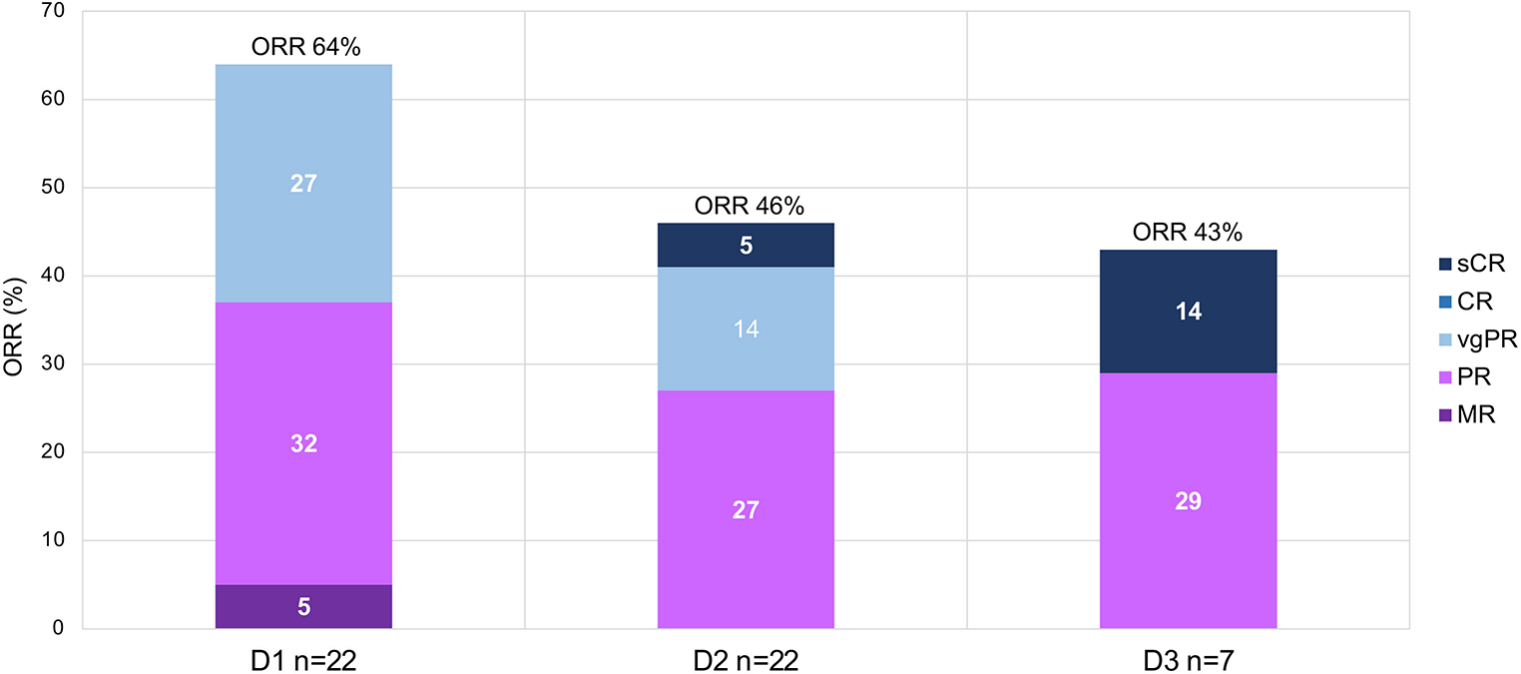
Best responses to daratumumab treatment in respective daratumumab lines. *Abbreviations*: ORR: overall response rate, sCR: stringent complete remission, CR: complete remission, vgPR: very good partial remission, PR: partial remission, MR: minor response, SD: stable disease, PD: progressive disease, ORR: overall response rate, D1 = first daratumumab treatment, D2 = first daratumumab retreatment, D3 = second daratumumab retreatment

**Fig. 3:**
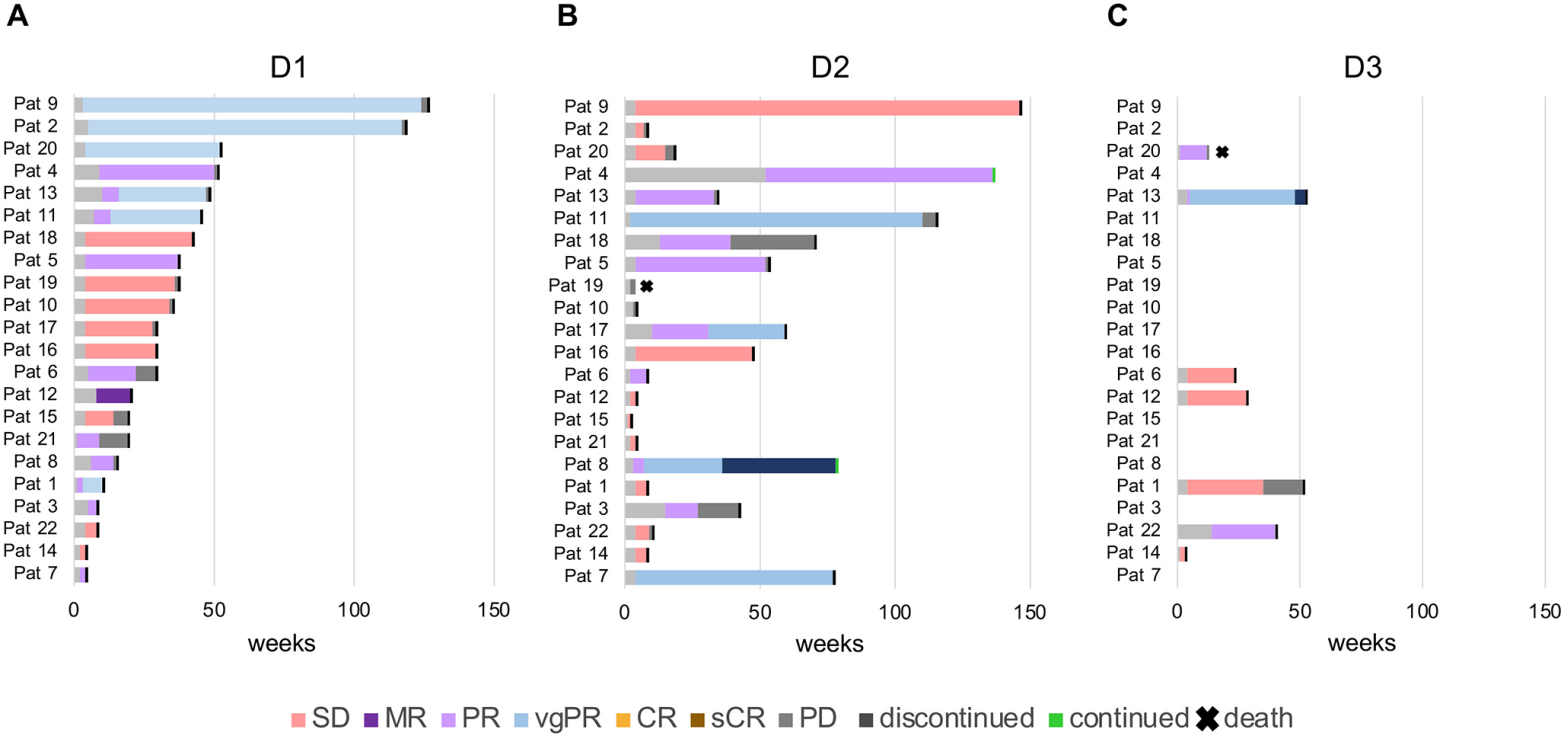
Direct comparison of all daratumumab lines, sorted according to the length of the first daratumumab treatment: (**A**) first daratumumab treatment (D1): response in 14 patients (67%), (**B**) first daratumumab retreatment (D2): response in 10 patients (46%) and second daratumumab retreatment (D3): response in 3 patients (43%) (response = MR or better). *Abbreviations*: SD: stable disease, PD: progressive disease, MR: minor response, PR: partial remission, vgPR: very good partial remission, sCR: stringent complete remission

The ORR was highest for D1 with 64%, followed by D2 with 46% and D3 with 43%. Best responses with stringent complete remission (sCR) occurred in one patient in D2 and D3 each. Very good partial remissions (vgPR) were achieved in 27% in D1 and in 14% in D2. Partial remissions (PR) were comparably achieved with 32% in D1, 27% in D2 and 29% in D3, and SD with 36% in D1, 45% in D2 and 57% in D3. Only two patients experienced progressive disease (PD) in D2 (9%) (Fig. 2; Table 2) Fig. 3 depicts swimmer plots for individual responses in each patient for D1, D2 and D3.

**Fig. 4:**
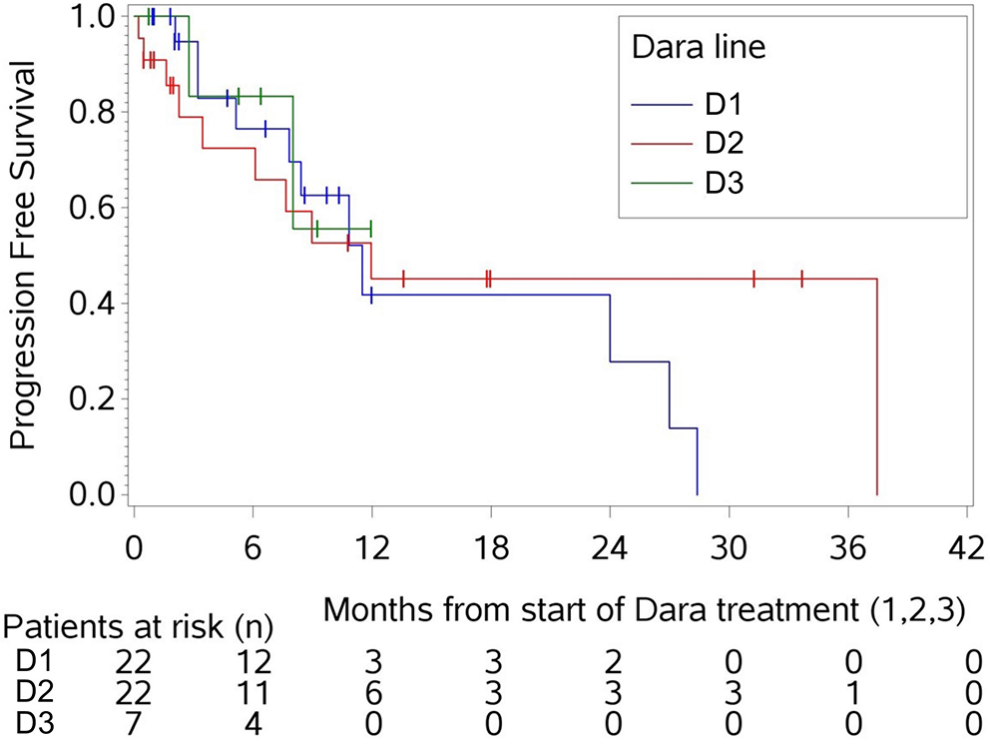
PFS of all daratumumab treatment-lines. Abbreviations: dara: daratumumab, D1 = first daratumumab treatment, D2 = first daratumumab retreatment, D3 = second daratumumab retreatment

Interestingly, the median PFS was comparable with 11.5 months (95% confidence interval [CI], 5.1–27) for D1, 12 months (95% CI: 3.4 - not estimable) for D2 and not reached (nr, 95% CI: 2.8 - not estimable) for D3. Thus, Kaplan-Meier curves of all daratumumab treatment-lines were superimposable (Fig. 4). In D1 the PFS rate at 12-months and 24-months decreased from 42% (95% CI: 13.2–70.3) to 28% (95% CI: 0-57.1), while in D2 this remained constant with 45% at both 12- and 24-months (95% CI: 20.1–70.2). The D3 cohort had a high 6-months PFS rate with 83% (95% CI: 53.5–100), 55.6% (95% CI 6.9–100) at 9 months; the 12 months PFS rate was outside the observation interval.

### Safety

Most patients had at least one treatment emergent (TE-) AE per daratumumab line (D1, D2 and D3) with 95%, 95% and 100%, respectively. Table 3 summarizes any grade TEAEs (≥ 57% in either group) that occurred during the different daratumumab treatment lines, with anemia being the most common TEAE in each daratumumab line. Of note, the incidence of overall concomitant cytopenias per daratumumab line were similar. Thus, anemia was reported in 17 patients (85%) in D1, in 20 patients (95%) in D2 and in 7 patients (100%) in D3. Leukopenias per daratumumab line were also very similar with 65%, 67% and 71%, respectively. Thrombocytopenia occurred in approximately ~ 70% of patients in D1 and D2 as compared to 57% in D3.

**Table 3:** Treatment-emergent adverse events (TEAE)

Event *n* (%)	First daratumumab treatment (D1, *n* = 20*)	1. daratumumab retreatment (D2, *n* = 21*)	2. daratumumab retreatment (D3, *n* = 7)
Hematologic			
**Anemia**			
Any Grade	17 (85)	20 (95)	7 (100)
Grade 1	10 (50)	8 (38)	1 (14)
Grade 2	2 (10)	7 (33)	2 (29)
Grade 3	5 (25)	5 (24)	4 (57)
**Leukopenia**			
Any Grade	13 (65)	14 (67)	5 (71)
Grade 3/4	5 (25)	5 (24)	3 (43)
**Thrombopenia**			
Any Grade	14 (70)	15 (71)	4 (57)
Grade 3/4	4 (20)	4 (19)	3 (43)
**Nonhematologic**			
**Infection**	7 (32)	4 (18)	2 (29)

* Insufficient data were available from some patients, this is why no hematological events could be determined here

Only the severity of TEAEs gradually increased, especially in D3 (Table 3). While the distribution of anemia in D1 from grade 1–3 was 50%, 10% and 25%, respectively, the number of grade 2 anemia in D2 increased to 33% with less patients showing grade 1 anemia (38%). In D3 most patients had grade 3 anemia with 57%. Leukopenia of grade 3/4 only occurred in ~ 25% of patients in D1 and D2 but increased in D3 (25%, 24%, 43%, respectively). Distribution of grades of thrombocytopenia was very similar in all daratumumab lines and grade 3/4 appeared in ~ 20% of patients in D1 and D2 but increased in D3 (20%/ 19%/ 43%, respectively). In all daratumumab lines, < 35% of all patients had an infection related to daratumumab administration (32% in D1, 18% in D2 and 29% in D3).

## Discussion

The results of our study suggest promising efficacy and highlighted the safety of daratumumab retreatment. Our total cohort represented a typical clientele of tertiary centers with increased age and advanced risk scores (ISS/ R-ISS/ R-MCI). Most had already received intensive anti-myeloma treatment with ≥ 3 lines of therapy with a substantial need to derive benefit from new therapeutic strategies.

Twenty-two patients (8%) of our primary MM-cohort receiving initial daratumumab were retreated (D2), with 7 patients even obtaining a second daratumumab retreatment (D3). Daratumumab showed high efficacy in all treatment lines, as evidenced by a favorable ORR with response declining only gradually from D1 (64%), to D2 (46%) and D3 (43%). Additionally, one patient each in D1 and D2 showed best response with sCR and all daratumumab lines showed consistent numbers of PRs with ~ 30%. Nooka et al. also analyzed ORR in daratumumab retreatment but compared daratumumab and pomalidomide-naïve patients (*n* = 12) with daratumumab- and/ or pomalidomide-refractory patients (*n* = 22) with similiar results (46% in our analysis and 41% in their analysis, respectively) [24]. In line, Abdallah et al. also found similar ORRs with 65% in D1 and 49% in D2 [21]. 

Superimposable PFS-curves of all daratumumab lines and comparable median PFS of D1 as compared to D2 and D3 (11.5, 12 months and nr, respectively) highlighted the efficacy of daratumumab retreatment. To be able to acquire a comparison group from other studies, we divided our total cohort into subgroups, first with groups of daratumumab combination partners. The most common combination partners were PIs (72% in D1, 41% in D2 and 57% in D3), followed by IMiDs. Then we divided them according to the lead substance with bortezomib being used most frequently (64% in D1, 27% in D2 and 14% in D3) and in detail with domination of Vd (45% in D1, 18% in D2 and 14% in D3). We also assessed in which median line of therapy daratumumab was given (2 in D1, 4 in D2 and 5 in D3) and searched for comparable literature that examined the PFS of Vd in the same line (Table 4). The ENDEAVOR trial (carfilzomib (K-)d vs. Vd) reported a PFS of 10 months for patients with 1 prior therapy line and 8.4 months with ≥ 2. [25] The CASTOR-trial (daratumumab (D-) Vd vs. Vd) showed a median PFS of 8 months (1 prior line) and 7.3 months (1–3 prior lines) [26]. Lu et al. in the LEPUS-trial (DVd vs. Vd in Chinese patients) described PFS of Vd after one prior line with 6.3 months [27]. Our PFS-rates with 11.5 months after one previous LOT and 12 months after 3 prior LOTs suggest superiority with daratumumab, combined with Vd (Table 4). This highlights the fact that there is effective synergism between daratumumab and its combination partner. Fully recognizing the limitations of comparing entirely different studies, rather than having a comparison group from our own cohort, we thus aimed to derive a comparison group as best as possible. Larger controlled trials are required to confirm the PFS of different combination partners of each daratumumab line of initial treatment and retreatment.

**Table 4:** Comparison groups: Outcome data of Vd in specific lines of therapy

Studies	Treatment	Median LOT (range)	PFS [mos]	ORR [%]	sCR [%]	vgPR [%]	PR [%]
Our analysis	First daratumumab treatment (D1)	2 (1–4)	11.5	64	0	27	32
	First daratumumab retreatment (D2)	4 (2–6)	12	46	5	14	27
ENDEAVOR (Kd vs. Vd)	Vd group	1 prior line	10.1	66	3	23	35
		≥ 2 prior lines	8.4	60	1	22	33
CASTOR (DVd vs. Vd)	Vd group	1 prior line	7.9	74	5	28	32
		1–3 prior lines	7.3	63	3	29	34
LEPUS (DVd vs. Vd)	Vd goup	1 prior line	6.3	68	-	-	-

*Abbreviations* Vd: bortzomib-dexamethasone, LOT: line of therapy, ORR: overall response rate, sCR: stringent complete remission, vgPR: very good partial response, PR: partial remission, PFS: progression free survival, mos: months

Of note, median treatment duration of all daratumumab lines was similar with 35, 42 and 30 weeks (9, 11, 8 daratumumab cycles) respectively, proving the effectiveness of daratumumab retreatment.

Patient characteristics of the daratumumab retreatment cohort would not suggest enrichment of low-risk myeloma. However, there may be a selection of patients with a rather slowly progressing disease and without early emergence of resistance to therapy, since the median time intervals between D1/D2 and D2/D3 are rather long with 34 and 32 weeks, respectively. In addition, a substantial part of the patients received no intercurrent therapy with 13/22 and 5/7 patients, respectively.

The occurrence of TEAEs were also similar in all daratumumab treatment lines demonstrating that there was no increased toxicity between daratumumab/CD38-antibody-lines. The most common cytopenia was anemia in all daratumumab lines, equally followed by leukopenia and thrombopenia. Grade 3–4 cytopenias were represented in D1 and D2 with less than 25%. Higher numbers in D3 may be explained with the advanced disease rather than being caused by the retreatment itself. Nooka et al. showed similar values, but focused only on daratumumab retreatment in combination with pomalidomide [24]. The safety profile of other studies, which examined the safety of daratumumab in combination with different therapeutic regimens, such as POLLUX (DRd vs. Rd) or CASTOR, was overall more favorable with less low-grade AEs, but the occurrence of AEs ≥ grade 3 was comparable to our study [28, 29]. A possible explanation could be that our cohort had undergone more intensive pre-treatment. In addition, the higher occurrence of only low-grade cytopenia suggests that they are clinically well-controllable events without any unexpected life-threatening risk. Treatment of infections occurred only in less than a third in the different daratumumab lines.

The strengths of our study are the rigorous analysis of real-world patients retreated with daratumumab in our department and our effort to compare the data to control groups from clinical trials in the literature. Limitations of this study were our number of patients due to the monocentric setting and the retrospective analysis, which lacked a control arm. Thus, confirmatory trials are necessary and suitable control groups to compare the PFS-rates of different combinations partners. In this retrospective analysis, no analyses according to the CD38-expression and wash-out periods were possible, because of insufficient samples. Nevertheless, Nijhof et al. and Perez de Acha et al. have already shown, that a wash out period allowed the re-expansion of myeloma cells with high CD38-expression, which leads to deeper responses [30, 31]. 

In summary, well-selected MM patients may show rewarding response to daratumumab/CD38-antibody-retreatment, the more with different anti-myeloma combination drugs, initial good response and CD38-antibody treatment pauses, proving this retreatment as feasible, effective and non-toxic. Until prospective trial data become available, our study may help to consider daratumumab retreatment as a possible treatment option in selected patients, i.e. those requiring response after chimeric antigen receptor (CAR)-T or bispecific antibody drug exposure.

## Data Availability

The data that support the findings of this study are available from the corresponding author (RW) upon reasonable request.
